# A human neuronal model of Niemann Pick C disease developed from stem cells isolated from patient’s skin

**DOI:** 10.1186/1750-1172-8-34

**Published:** 2013-02-21

**Authors:** Natascha Bergamin, Andrea Dardis, Antonio Beltrami, Daniela Cesselli, Silvia Rigo, Stefania Zampieri, Rossana Domenis, Bruno Bembi, Carlo Alberto Beltrami

**Affiliations:** 1 Department of Medical and Biological Sciences (DSMB), University of Udine, P.le Kolbe 3, 33100, Udine, Italy; 2 Regional Coordinator Centre for Rare Diseases, University Hospital “Santa Maria della Misericordia”, Udine, Italy

**Keywords:** Niemann Pick C, Human neuronal model, Adult stem cells, Neuronal differentiation

## Abstract

**Background:**

Niemann Pick C (NPC) disease is a neurovisceral lysosomal storage disorder due to mutations in *NPC1* or *NPC2* genes, characterized by the accumulation of endocytosed unesterified cholesterol, gangliosides and other lipids within the lysosomes/late endosomes. Even if the neurodegeneration is the main feature of the disease, the analysis of the molecular pathways linking the lipid accumulation and cellular damage in the brain has been challenging due to the limited availability of human neuronal models.

**Objective:**

The aim of this study was to develop a human neuronal model of NPC disease by inducing neuronal differentiation of multipotent adult stem cells (MASC) isolated from NPC patients.

**Methods:**

Stem cells were isolated from 3 NPC patients and 3 controls both from skin biopsies and previously established skin fibroblast cultures. Cells were induced to differentiate along a neuronal fate adapting methods previously described by Beltrami et al, 2007. The surface immunophenotype of stem cells was analyzed by FACS. Stem cell and neuronal markers expression were evaluated by immunofluorescence. Intracellular accumulation of cholesterol and gangliosides were assessed by filipin staining and immunofluorescence, respectively. A morphometric analysis was performed using a Neurite outgrowth image program.

**Results:**

After 3 passages in selective medium, MASC isolated either from skin biopsies or previously established skin fibroblast cultures displayed an antigenic pattern characteristic of mesenchymal stem cells and expressed the stem cell markers Oct-4, Nanog, Sox-2 and nestin. A massive lysosomal accumulation of cholesterol was observed only in cells isolated from NPC patients. After the induction of neural differentiation, remarkable morphologic changes were observed and cells became positive to markers of the neuronal lineage NeuN and MAP2. Differentiated cells from NPC patients displayed characteristic features of NPC disease, they showed intracellular accumulation of unesterified cholesterol and GM2 ganglioside and presented morphological differences with respect to cells derived from healthy donors.

In conclusion, we generated a human neuronal model of NPC disease through the induction of differentiation of stem cells obtained from patient’s easily accessible sources. The strategy described here may be applied to easily generate human neuronal models of other neurodegenerative diseases.

## Introduction

Niemann Pick C [NPC-MIM 257220; MIM607625] disease is a neurodegenerative lysosomal storage disorder due to mutations in *NPC1* or *NPC2* genes, characterized by the accumulation of endocytosed unesterified cholesterol, gangliosides and other lipids within the lysosomes/late endosomes. Both proteins are involved in the intracellular trafficking of cholesterol and other lipids. Thus, the deficiency of either of them leads to the accumulation of the endocytosed unesterified cholesterol, gangliosides and other lipids within the lysosome/late endosome compartment [1].

Clinically, NPC disease presents a highly variable phenotype ranging from fetal to adult age. Even though initial manifestations are typically systemic, including liver and spleen enlargement, the disease has been classified according to the age at onset of neurological symptoms in: severe infantile form (onset before 2 y of age), late infantile form (onset between 3-5 y of age), juvenile form (onset between 5 and 16 y) and adult form (onset at age>16 y) [1,2].

Approximately 95% of NPC patients present mutations in *NPC1* gene (MIM 607623; chr 18q11-q12) [3,4], which encodes a membrane glycoprotein of 1,278 amino acids containing 13 transmembrane domains and localized in late endosomes [5]. The other 5% of patients present mutations in *NPC2* gene (MIM 601015; chr 14q24.3) [6] encoding a soluble 151 amino acid protein that is present in the lumen of lysosomes.

Despite the progress in characterizing the biochemical and genetic defects in NPC disease, the mechanisms underlying the pathophysiology of this disorder are not clear and the currently available therapeutic interventions are limited. In particular, the analysis of the molecular pathways linking the lipid accumulation and cellular damage in the brain has been challenging due to the limited availability of neuronal models.

Two mouse models of NPC disease have been described and used to study NPC pathogenesis, the BALB\c NPC [7] and the Npc1 (nmf164) mouse [8]. The naturally occurring BALB\c NPC mouse recapitulates the main features of human pathology [7]. However, while this model presents a very severe phenotype, most NPC patients present with a less severe form of the disease. This issue is particularly relevant when this model is used to test new potential therapies since the very acute nature of the BALB\c NPC mouse model may mask the potential benefits of therapies that could be useful in a clinical setting in patients. Recently, a new mouse model of NPC disease, Npc1 (nmf164) carrying a c.3163A>G mutation that results in an aspartate to glycine change at position 1005 (D1005G), has been generated. This mutant mouse displays a slower development of the NPC phenotype than the BALB\c NPC mouse. Therefore, it may represent a good model for the late-onset, slower progressing forms of NPC disease [8]. However, it is worth noting that some characteristic features of NPC human neurons are not present in mice, suggesting important species differences between mice and human NPC neurons [9-11].

Many studies have been performed in peripheral cells in culture. However, the obtained results might not be extrapolated to neuronal cells since the pattern of accumulated lipids is quite different between peripheral and central tissues [12].

Recent advances in human stem cell biology and the optimization of protocols for *in vitro* differentiation of stem cells into different cell lineages have opened new possibilities for generating disease cellular models.

Pluripotent cells have the ability to form all the body's cell lineages, including germ cells and some extra-embryonic cell types [13]. The analyisis of blastocyst chimerism and tetraploid aggregation followed by gestation are the most appropriate tests employed to assess mouse stem cell pluripotency [13]. Human stem cell pluripotency cannot be tested for their ability to generate fully functional germ cells *in vivo* without raising ethical concern. Consequently, a surrogate test for human stem cell pluripotency is teratoma formation upon administration to a receptive animal [13]. Interestingly, a number of reports have demonstrated that adult human tissues host widely multipotent cells. Although these latter are able to differentiate, both *in vitro* and *in vivo*[13,14], into mesodermal [15], ectodermal [16] and endodermal [17] derivatives, even at a clonal level [18], they do not form either teratomas *in vivo* or contribute to the germ cell lineages, thus not entirely fulfilling the criteria for pluripotency. This may be secondary to epigenetic differences between adult and embryonic stem cells [14].

Our group has described a highly reproducible method to isolate, from different adult human tissues (heart, liver and bone marrow), a population of multipotent stem cells (named Multipotent Adult Stem Cells or MASC), which express several pluripotent state specific transcription factors (i.e. OCT4, Nanog, Sox2, and Rex1), display high levels of telomerase activity and show all the characteristics of stem cells, such as clonogenicity and self renewal. Furthermore, they present a highly similar gene expression profile, irrespectively from the tissue of origin and they maintain the ability to differentiate along some derivatives of the 3 germ layers, including neuroectoderm [19].

The aim of this study was to develop a human neuronal model of NPC disease by inducing neuronal differentiation of stem cells isolated from skin biopsies or fibroblasts in culture obtained from NPC patients.

## Methods

### Human samples

Specimens from 3 patients affected by NPC disease (two of them were siblings) and 3 normal controls have been used in this study. Samples from a patient affected by Sandhoff disease were used as positive control for ganglioside accumulation assays.

All 3 NPC patients presented the classical biochemical phenotype characterized by massive lysosomal/late endosomes accumulation of unesterified cholesterol in cultured fibroblasts. The diagnosis was confirmed by sequencing both *NPC1* and *NPC2* genes. All patients had mutations in the *NPC1* gene. The two affected siblings presented the c.3182T>C (P.I1061T)/c.3182T>C (p.I1061T) genotype, while the third unrelated NPC patient presented the c.2795+1G>C/c.3493G>A (p.V1165M) genotype. This study was approved by the ethical committee of the University Hospital “S. Maria della Misericordia” and written consent was obtained from all subjects.

### Stem cell selection and culture

Stem cell enriched cultures were obtained, both from skin biopsies and from already established skin fibroblast cultures at early passages (P1, P2, P3), adapting the methods previously described [19,20].

Briefly, skin biopsies were minced and digested with 0.04% of collagenase type I (Sigma-Aldrich) for 15 minutes at 37°C. Cell suspension was filtered through a 40 μm nylon membrane (Dako) and 4 × 10^4^ cells/mm^2^ were plated onto fibronectin coated dishes (10 μg/100 mm plate, from Sigma-Aldrich). In alternative, 2 × 10^6^ cells directly isolated from human skin biopsies or 1 × 10^6^ cells obtained from confluent primary skin fibroblast cultures, were seeded onto 100 mm plates coated with fibronectin and expanded at least for three passages in a selective media composed of 60% Dulbecco’s Modified Eagle medium (DMEM)/40% MCDB-201 (Sigma-Aldrich) supplemented with 1 mg/ml Linoleic Acid-BSA (Sigma-Aldrich);10^-9^ M dexamethasone (Sigma-Aldrich); 10^-4^ M Ascorbic acid-2 phosphate (Sigma-Aldrich); 1X Insulin-transferrin-sodium selenite (Sigma-Aldrich); 2% fetal bovine serum (FBS), (STEMCELL Technologies), 10 ng/ml human PDGF-BB (Peprotech EC); 10 ng/ml human EGF (Peprotech EC). Medium was replaced every 4 days and cells were split when they reached 70/80% confluence.

### Single cell cloning

Stem cells, at the third passage in culture (P3), obtained from healthy donors (n = 600 cells) and NPC patients (n = 600 cells) were individually deposited directly into fibronectin-coated wells of 96-well plates (Falcon, BD-Biosciences, Italy) with an automated cell sorter (FACSAria, BD), and cultured in expansion medium supplemented with 10% FBS [19,20]. To determine sorting efficiency and to verify if any well was seeded with more than one cell, we utilized the Vybrant CFDA SE (CFSE) as a cell tracker (Molecular Probes, Invitrogen). Wells were examined twice a week in order to determine the fraction of cells able to give rise to proliferating clones.

### Multilineage differentiation

Multilineage differentiation was evaluated as previously described [19,20].

Muscle cell- and endothelial cell- differentiation was achieved plating 0.5 to 1 × 10^4^/cm^2^ cells in expansion medium containing 5% FBS (Sigma-Aldrich), 10 ng/mL bFGF, 10 ng/mL VEGF, and 10 ng/mL IGF-1 (all from Peprotech EC), but not EGF. Cells were allowed to become confluent and cultured for up to 2 weeks with medium exchanges every 4 days.

Hepatocytic differentiation was induced growing cells for two weeks at high density (2 × 10^4^/cm^2^) onto fibronectin coated coverslips in a medium containing 0.5% FBS, 10 ng/ml FGF-4 and 20 ng/ml HGF (both from Peprotech EC).

For neurogenic differentiation, stem cells obtained after 3 passages in selective medium, were seeded at a density of 8000 cells/cm^2^ into 96 multiwell plates (BD Biosciences) or on coverslips. The differentiation protocol was adapted from a method previously described [19,20]. Briefly, cells were plated in medium containing DMEM-HG with 10% FBS (called N1 medium). After 24 hours the DMEM-HG was replaced with fresh medium supplemented with 1% of B27 (Invitrogen), 10 ng/ml EGF (Peprotech) and 20 ng/ml bFGF (Peprotech) (called N2 medium) for 5 days. Thereafter, cells were incubated for 24/48 hours in DMEM supplemented with 5 μg/ml insulin, 200 μM of indomethacin and 0.5 mM IBMX (all from Sigma-Aldrich) without FBS (called N3 medium).

### Flow cytometry analysis

After at least 3 passages in selective medium, stem cells were detached and stained with the following primary conjugated antibodies: CD10, CD13, CD29, CD49a, CD49b, CD49d, CD90, CD73, CD44, CD45, HLA-DR, CD117, CD34, CD271 (BD Biosciences), CD105, CD66e, KDR (Serotech), CD133 (Miltenyi Biotec), CXCR4, (R&D), ABCG-2 (Chemicon International). The percentage of cells expressing all considered antigens was determined by flow cytometry analysis (CyAn, Beckman Coulter). Properly conjugated isotype matched antibodies were used as negative controls.

### Filipin staining

Filipin staining was performed using the method described by Blanchette-Mackie et al. [21]. Briefly, cells grown on coverslips, were incubated in serum free medium for 24 hours and then treated for 24 hours with LDL enriched medium. Cells were rinsed with PBS and fixed with 3% paraformaldehyde. After washing them with PBS, the cells were incubated with 1.5 mg of glycine/ml PBS for 10 minutes, stained with filipin (0.05 mg/ml, in PBS 10% FCS) for 2 hours and examined using a Zeiss fluorescence microscope.

### Periodic acid Schiff staining (PAS)

PAS was employed to detect glycogen accumulation. Slides were oxidized in 1% periodic acid for 5 minutes, rinsed three times in distilled water and treated with Schiff’s reagent for 25 minutes. After extensive washing, slides were stained with Mayer’s hematoxylin for 10 minutes.

### Immunofluorescence microscopy

*Protein markers*: Cells were grown on coverslips then fixed in 4% paraformaldehyde for 20 minutes at room temperature, permeabilized 10 minutes at room temperature with 0.1% Triton X-100 (Sigma-Aldrich) and stained overnight at 4°C to visualize stem cell markers: Oct4 (Abcam, Rabbit polyclonal, 1:150), Nanog (Abcam, Rabbit polyclonal, 1:200), Sox2 (Millipore, Mouse monoclonal, 1:200), Nestin (Millipore, 1:100); myocytes specific markers: smooth muscle actin (SMA) (Sigma, 1:50 mouse monoclonal) and α-sarcomeric actin (ASA) (Sigma, 1:100 mouse monoclonal); endothelial cell marker: CD31 (Dako, 1:50 mouse monoclonal); hepatic specific marker: cytokeratins 8, 18, and 19 (CK) (1:50 mouse monoclonal); or neural specific markers: tubulin beta 3 (COVANCE, 1:1000 mouse monoclonal), NeuN, (Millipore, 1:50 mouse monoclonal) and MAP2 (Millipore, 1:50 rabbit polyclonal). Secondary antibody staining was done with donkey anti-rabbit or donkey anti-mouse antibodies (Alexa-Fluor 555 or 488, Molecular Probes) at 1:600 dilutions. Images were obtained with a live cell imaging dedicated system consisting of a Leica DMI 6000B microscope connected to a Leica DFC350FX camera (Leica Microsystems); 10X (numerical aperture: 0.25), 40X oil immersion (numerical aperture: 1.25) and 63X oil immersion (numerical aperture: 1.40) objectives were employed.

*GM2andGM3analysis*: The analysis of GM2 and GM3 gangliosides was performed as previously described [22]. Cells were grown on coverslips then fixed in 4% paraformaldehyde for 45 minutes at room temperature. Cells were then incubated at room temperature for 1 hour in blocking buffer [PBS with 10% normal donkey serum (NDS) and 0.02% saponin (Sigma-Aldrich)] and stained overnight at 4°C with mouse anti-GM2 (1:20 in blocking buffer) or mouse anti-GM3 (1:20 in blocking buffer). Cells were then incubated with a TRITC conjugated donkey anti-mouse IgM [1:80 in PBS with 2% NDS and 0.02% saponin (Sigma-Aldrich)]. For colocalization studies, a monoclonal anti-LIMP-1 (Novus Biologicals, Littleton, USA) was used as a primary antibody, and an Alexa fluor-conjugated anti-mouse (Invitrogen, Carlsbad, CA, USA) as a secondary antibody.

In all cases, nuclei were stained by DAPI (Vector Laboratories, Inc) and Vectashield (Vector) was used as mounting medium. Epifluorescence and phase contrast images were obtained with a live cell imaging dedicated system consisting of a Leica DMI 6000B microscope connected to a Leica DFC350FX camera (Leica Microsystems); 10X (numerical aperture: 0.25), 40X oil immersion (numerical aperture: 1.25) and 63X oil immersion (numerical aperture: 1.40) objectives were employed. Adobe Photoshop software was utilized to compose and overlay the images, and adjust contrast (Adobe, USA). The counts of positive cells were done manually, considering, where possible, 100 positive events per sample.

### Real-time RT-PCR

Total RNA was extracted from both non-confluent cultures of undifferentiated and differentiated cells at P3 using the TRIzol Reagent (Invitrogen). After treatment with DNase I (Ambion), first strand cDNA synthesis was performed with 1 μg total RNA using random hexanucleotides and MMLV reverse transcriptase (Invitrogen). Primers were designed from available human sequences using the primer analysis software Primer3 (Additional file 1: Table S1). Quantitative RT-PCR was performed using Roche LightCycler 480 Real-Time PCR System and the LightCycler 480 SYBR Green I Master (Roche), following manufacturer’s instructions. GAPDH was used as internal control for normalization. LightCycler 480 Basic software (Roche) utilized the second derivative maximum method to identify the crossing point (Cp).

### Evaluation of apoptosis

Apoptosis in differentiated cells at P3 was evaluated by staining of phosphatidylserine exposed on cell membranes with FITC labeled Annexin V, according to the manufacturer’s instructions (Sigma-Aldrich) and analyzed by flow cytometry using a FACScan (Becton Dickinson, Franklin Lakes, NJ, USA).

### Morphological analysis

Morphological analysis data were collected by using the BD Pathway bioimaging platform. Differentiated cells were first immunostained for the neural marker MAP-2, then images were acquired on a BD Pathway 855 using a 20X objective (0.75 NA) in the form of 2 × 2 montage. The images were then analyzed using BD’s Neurite Outgrowth Algorithm that automatically measure parameters describing neurite outgrowth.

### Statistical analysis

Statistical analysis was performed using Student’s *t* test or one-way ANOVA test, followed by Bonferroni post-test. The analyses were carried out using the software Prism, version 4.0c, GraphPad Software, San Diego, CA, USA; JMP7, SAS Institute Inc., Cary, NC, USA.

p<0.05 was considered statistically significant.

## Results

### Stem cell characterization

In order to isolate multipotent stem cells from NPC patient samples, we applied a protocol that was optimized for the growth of widely multipotent cells with mesenchymal features (named human multipotent adult stem cells-hMASC) from several human tissues [19,20] to both freshly obtained skin biopsies and previously established skin fibroblast cultures. If successful, this latter strategy would allow us to derive multipotent cells from already available bio-repositories of cell lines, obtained from patients suffering from rare diseases, such as NPC, for diagnostic purposes.

**Figure 1:**
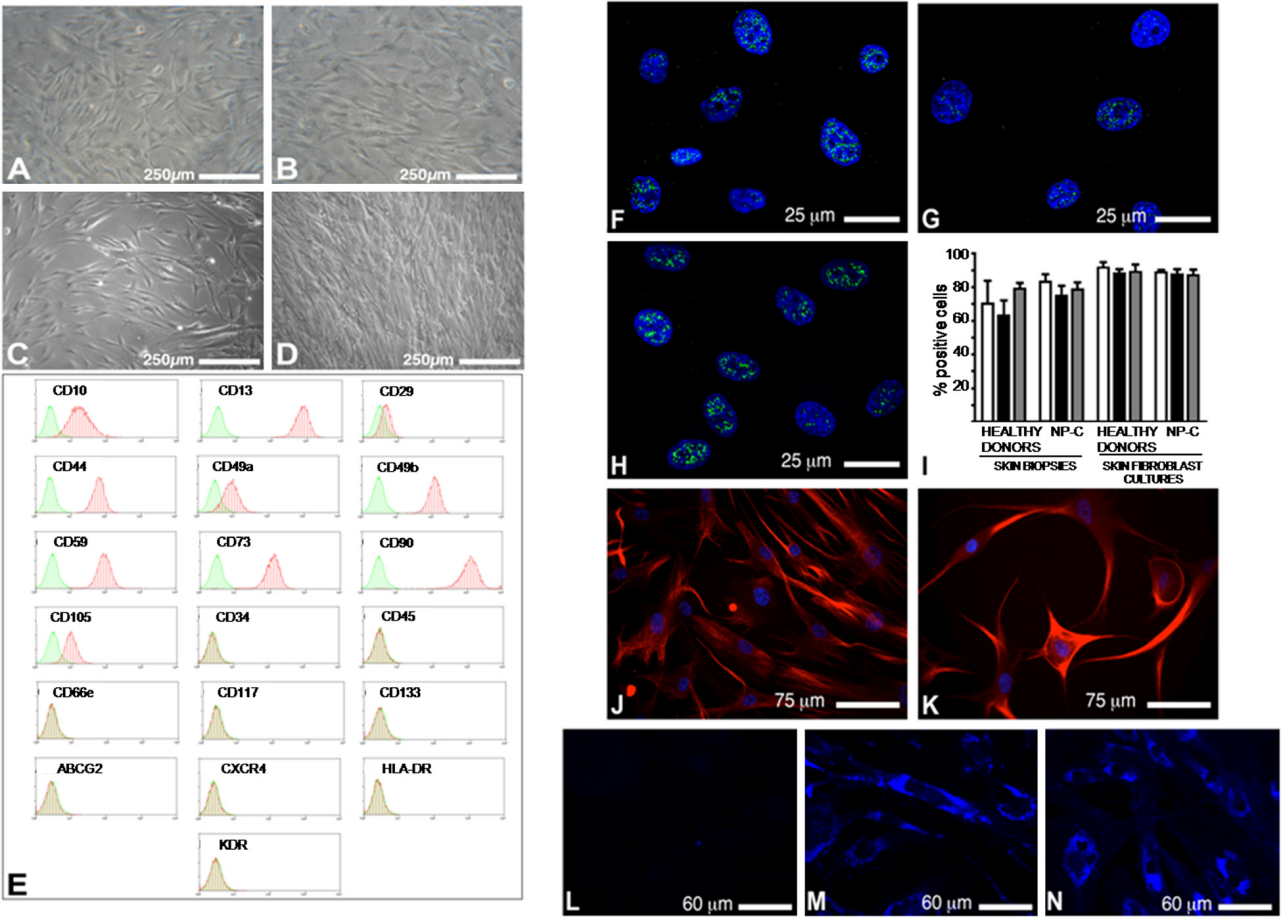
**Characterization of stem cell populations obtained from skin biopsies or already established skin fibroblast cultures.** (**A**-**D**) Phase contrast images of hSKIN-MASC at the third passage in culture: **A**-**B**) hSKIN-MASC obtained from skin biopsies of a healthy donor (**A**) and a NPC patient (**B**). **C**-**D**) hSKIN-MASC obtained from already established skin fibroblasts from healthy donor (**C**) and NPC patient (**D**). (**E**) Surface immunophenotype: representative flow cytometry histograms of skin derived stem cell cultures. Plots show isotype control IgG-staining profile (green histograms) versus specific antibody staining (red histograms). (**F**-**H**) Pluripotent state specific transcription factor expression: representative fluorescence images of Oct-4 (green fluorescence; **F**), Nanog (green fluorescence; **G**) and Sox-2 expression (green fluorescence; **H**) localized in the nuclei of skin derived stem cell cultures. Nuclei are depicted by the blue fluorescence of DAPI staining (**F-H**). **I)** Quantification of the percentage of cells expressing Oct4 (white bars), Nanog (black bars) and Sox-2 (gray bars) in hSKIN-MASC obtained both from healthy donors (CTRL, n = 3) and NPC (n = 3) skin biopsies or healthy donors (CTRL, n = 3) and NPC (n = 3) skin fibroblast cultures. At least 400 cells have been counted for each cell line. Data are presented as mean ± SD of 3 independent experiments. (**J**-**K**) Nestin expression (red fluorescence) in hSKIN-MASC obtained from a healthy donor (**J**) and a NPC patient (**K**). (**L**-**N**) Unesterified cholesterol accumulation: images obtained after performing the filipin staining (blue fluorescence) in hSKIN-MASC derived from a healthy donor skin biopsy (**L**), a NPC patient skin biopsy (**M)** and a NPC skin fibroblast cell line (**N**)**,** respectively.

When exposed to a selective culture medium enabling the growth of hMASC, proliferating cell lines could be obtained from all skin biopsies. On the contrary, only those fibroblast cultures that had not been extensively expanded *in vitro* (< 3 passages *in vitro*) were responsive to these stringent culture conditions. After 3 passages in the selective medium, the cells acquired a homogeneous morphology as shown in Figure 1A-D. No differences were observed neither between cells obtained from NPC patients and normal controls nor between cells obtained from biopsies or from already established skin fibroblast cultures.

In order to characterize the selected cells, their surface immunophenotype was analyzed. As shown in Figure 1E and Table 1, cells obtained both from skin biopsies and fibroblast cell lines displayed an antigenic pattern characteristic of mesenchymal stem cells, very similar to the one observed in MASC isolated from heart, liver or bone marrow [19]. No major differences were observed between cells derived from NPC patients or normal controls.

**Table 1:** Surface immunophenotype of stem cells isolated from skin biopsies or culture fibroblasts

	**Healthy donor (skin biopsy)**	**NP-C (skin biopsy)**	**Healthy donor (fibroblast culture)**	**NP-C (fibroblast culture)**
**CD49d**	95.9 ± 3.2	99.89 ± 0.22	99.1 ± 0.7	99.9 ± 0.08
**CD49a**	37.5 ± 7.2	48.6 ± 0.4	6.38 ± 2.51	54.18 ± 3.44
**CD49b**	96.48 ± 2.8	99.21 ± 0.3	99.98 ± 0.1	99.95 ± 0.2
**CD29**	43.33 ± 12.5	62.75 ± 1.05	17.05 ± 14.7	17.62 ± 4.9
**ABCG2**	1.57 ± 0.7	1.14 ± 0.02	1.81 ± 0.06	1.37 ± 0.25
**CXCR4**	0.22 ± 0.1	0.16 ± 0.01	0.081 ± 0.06	0.18 ± 0.05
**CD10**	77.8 ± 13	98 ± 0.4	55.5 ± 20.7	96.05 ± 1.2
**CD66**	1.23 ± 0.7	3.04 ± 0.05	0.72 ± 0.7	0.28 ± 0.23
**CD133**	2.06 ± 0.34	5.88 ± 0.28	0.31 ± 0.08	0.23 ± 0.12
**CD271**	2.95 ± 1.33	0.2 ± 0.071	0.37 ± 0.31	0.89 ± 0.24
**CD44**	95.89 ± 2.7	97.25 ± 0.2	99.41 ± 0.7	98.94 ± 1.02
**CD105**	87 ± 3.4	85.9 ± 2.78	92.73 ± 4.35	74.92 ± 30.8
**CD90**	97.12 ± 3.2	98.88 ± 1.41	99.52 ± 0.28	96.25 ± 3.7
**CD73**	92.9 ± 3.7	99.75 ± 0.08	99.48 ± 0.4	99.7 ± 0.19
**CD34**	3.7 ± 1.7	1.9 ± 0.1	0.205 ± 0.12	0.66 ± 0.83
**CD45**	0.1 ± 0.2	0.19 ± 0.01	0.45 ± 0.04	0.07 ± 0.02
**KDR**	3.12 ± 1.1	1.47 ± 0.03	1.92 ± 2.18	1.53 ± 0.18
**CD13**	98.49 ± 3.7	98.4 ± 1.9	99.78 ± 0.21	99.88 ± 0.15
**CD117**	1.1 ± 0.67	0.52 ± 0.03	0.15 ± 0.13	0.27 ± 0.3
**HLA-DR**	0.37 ± 0.1	0.12 ± 0.14	0.65 ± 1.01	0.03 ± 0.01

Cultured cells were then evaluated for the expression of stem cell markers, such as the pluripotent state specific transcription factors Oct-4, Nanog and Sox-2 and the intermediate filament nestin. As shown in Figure 1F-K, the vast majority of cells (65 to 90%) expressed these markers and this expression was independent from the disease state. In fact, no significant differences were observed between NPC and normal cells or between cells isolated from both skin biopsies and early passages of fibroblast cell lines.

In order to investigate whether the pathologic phenotype was retained in these cells, the intracellular accumulation of unesterified cholesterol was analyzed by filipin staining. A massive accumulation of cholesterol within the endosomal/lysosomal compartment was observed in cells isolated from either skin biopsies or fibroblast cultures of NPC patients (Figure 1L-N).

Therefore, in the light of these data, we decided to further characterize stem cells derived from already established skin fibroblast cultures and use them to develop a neuronal differentiation model.

**Figure 2:**
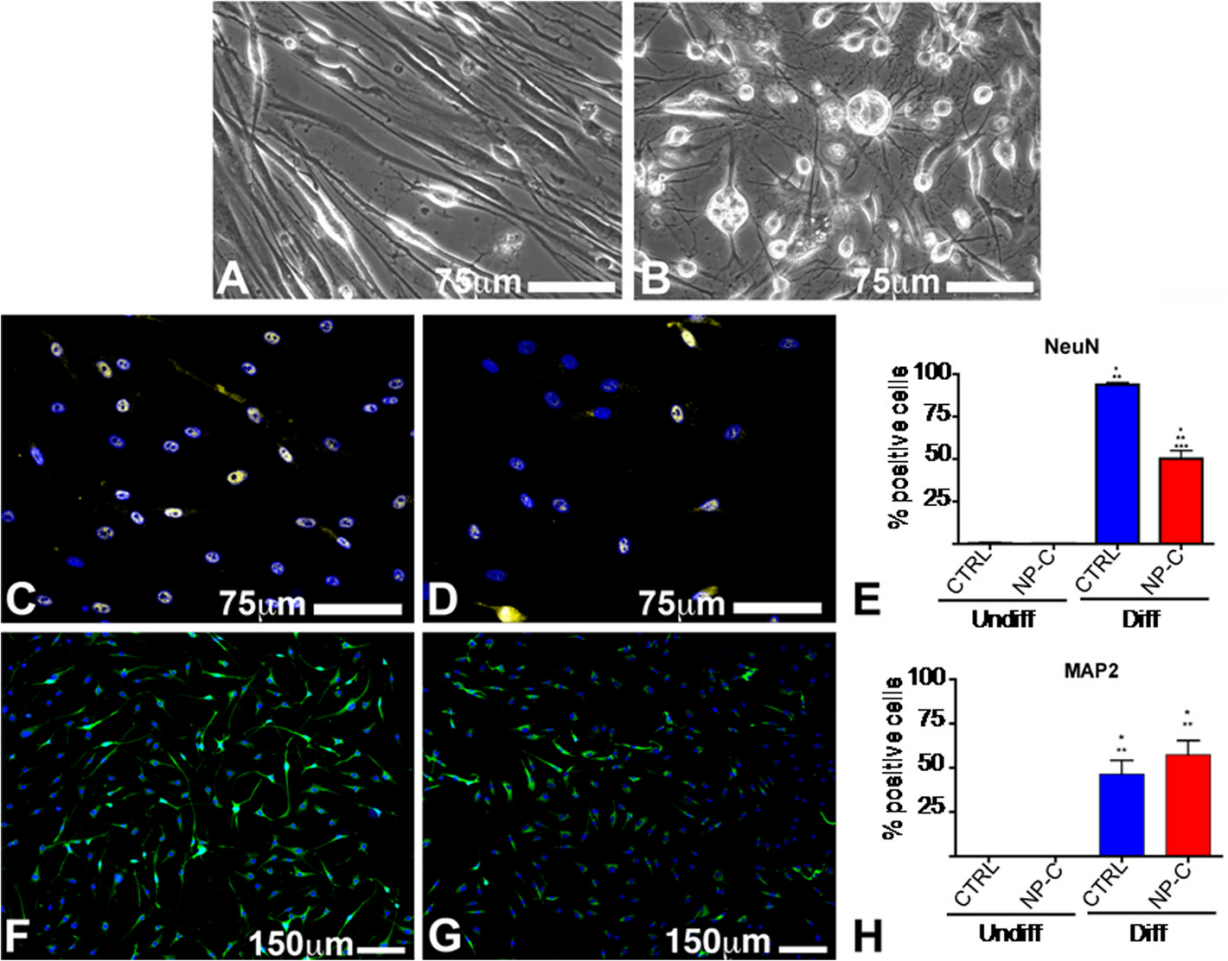
**Neural differentiation of hSKIN-MASC obtained from already established skin fibroblast cultures.** (**A-B**) Phase contrast images of hSKIN-MASC after induced to differentiate toward a neuronal fate (5 days in N2 medium + 48 h in N3 medium, see methods) from a healthy donor (**A**) and a NPC patient (**B)**. (**C-H**) Neuronal markers detection: differentiated cells (5 days in N2 medium + 48 h in N3 medium, see methods), obtained from a healthy donor (**C**, **F**) or a NPC patient (**D**, **G**), express the neuron specific markers NeuN (yellow fluorescence, **C**, **D**) and MAP2 (green fluorescence, **F**, **G**). Nuclei are depicted by the blue fluorescence of DAPI staining. Quantitative evaluation of the percentage of cells expressing NeuN (**E**) or MAP2 (**H**) in cultures from healthy donors (CTRL, n = 3) and NPC patients (n = 3) skin fibroblast derived cultures, before (Undiff.) and after the exposure to neuron induction media (Diff.). At least 400 cells have been counted for each cell line. Data are presented as mean ± SD of 3 independent experiments one-way Anova test followed by Bonferroni post-test were utilized to compare means between groups. P values less than 0.05 were considered significant. P values less than 0.05 were considered significant. *, **, ***, *p*<0.05 vs. columns 1, 2, and 3, respectively.

Cell cultures showed two of the major features of multipotent adult stem cells such as clonogenicity and wide differentiation capacity. Specifically, when sorted as single cells into the wells of 96-well Terasaki plates, skin-derived cells were able to form proliferating colonies within 2 weeks after seeding. It is worth noting that cells derived from NPC patients seem to be less clonogenic than cells derived from healthy controls. Even if the differences were not statistically significant, this result suggests that some stem cells features may be compromised in NPC disease. The cells were able to differentiate along derivatives of all the three germ layers (Figure 2, Additional file 2: Figure S1 and Additional file 3: Figure S2). In this regard, cultured cells generated not only neurectodermic derivatives (see below) but mesodermal and endodermal ones as well. Specifically, cells derived from both healthy controls and NPC patients cultured in a medium added with IGF-1, bFGF and VEGF expressed the myocyte specific markers alpha-sarcomeric actin (ASA) and smooth muscle actin (SMA) and the endothelial cell marker CD31 (Additional file 2: Figure S1). Additionally, cells exposed to hepatocyte differentiation medium assumed a globular shape and became positive for cytokeratins 8, 18, and 19 (CK). Moreover, they acquired some hepatocitic functions such as the ability to store glycogen as demonstrated by the PAS staining (Additional file 3: Figure S2).

Altogether these results indicate that cells obtained from fibroblast cultures of healthy controls and NPC patients exhibited almost an identical mesenchymal stem cell immunophenotype, expressed pluripotent state specific transcription factors, were clonogenic and multipotent; therefore, in analogy with our previous studies, we named them hSKIN-MASC.

### Neural differentiation

Twenty-four hours after the induction of neural differentiation, hSKIN-MASC showed remarkable morphologic changes. As shown in Figure 2 (panels A-B) differentiated cells displayed an enlargement of the cellular bodies and the presence of long projections, closely resembling the morphology of neuronal cells. Importantly, the morphology of differentiated cells obtained from NPC patients and healthy donors was clearly distinct. In fact, with respect to healthy donors (Figure 2A), differentiated cells obtained from NPC patients (Figure 2B) were larger and presented numerous projections similar to dendrites.

Upon differentiation, a large fraction of hSKIN-MASC became positive to markers of the neuronal lineage. In particular, they expressed NeuN (Figure 2C-E), a neuronal specific nuclear protein, and MAP2 (Figure 2F-H), a structural protein specifically present in neuronal cells. Interestingly, the percentage of cells expressing NeuN was significantly lower in cells derived from NPC patients than in cells derived from healthy donors. In addition, differentiated cells did not express markers associated with glial differentiation, such as glial fibrillary acidic protein (GFAP) and oligodendroglial protein 4 (O4) (data not shown). These data suggest that the differentiation protocol described here specifically favored the differentiation towards the neuronal lineage. It is worth noting that, after differentiation, the cells became negative for the pluripotent state specific transcription factors Oct-4, Nanog and Sox-2, while the expression of nestin was maintained throughout the differentiation process.

To further characterize the type of neuronal cells obtained, mRNA expression of specific markers of dopaminergic (tyrosine hydroxylase, TH and dopamine transporter, DAT), cholinergic (choline acetyltransferase, CHAT) and GABAergic (glutamic acid decarboxylase, GAD) neurons were analyzed by real time PCR. None of these markers were detected in undifferentiated cells. After differentiation, both cells derived from healthy donors and NPC patients expressed similar levels of CHAT mRNA (Additional file 4: Figure S3), while the levels of TH, DAT and GAD mRNA were undetectable.

No differences in the levels of apoptosis have been detected between differentiated cells derived from healthy donors and NPC patients (Additional file 5: Figure S4).

**Figure 3:**
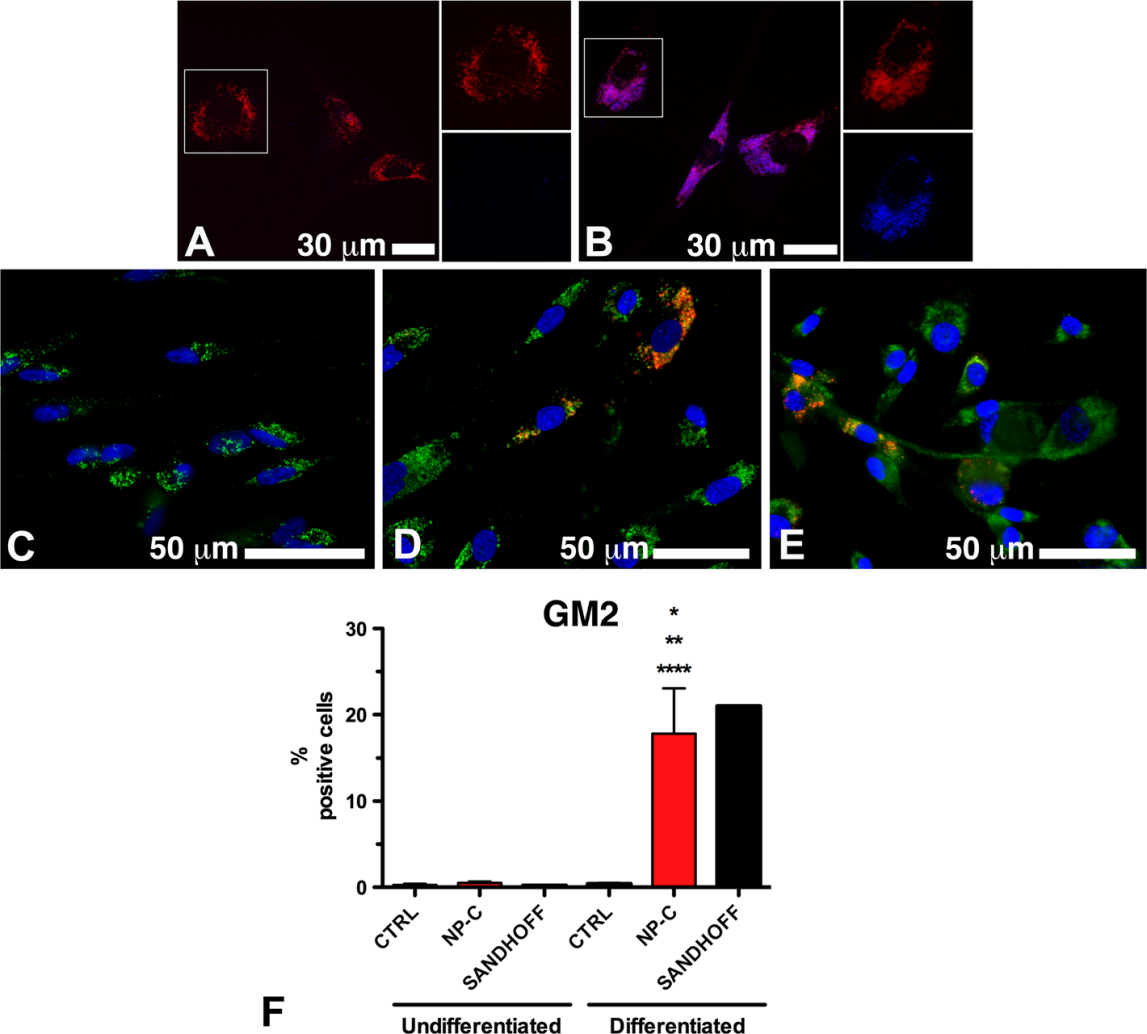
**Pathologic accumulation of unesterified cholesterol and GM2 in differentiated hSKIN-MASC derived from NPC patients.** (**A** -**B**) co-immunostaining for the lysosome marker Limp 1 (red fluorescence) and for unesterified cholesterol (blue fluorescence), revealed lysosomal accumulation of cholesterol after neuronal differentiation (5 days in N2 medium + 48 h in N3 medium, see methods) only in cultures derived from an NPC patient (**B**), but not in healthy donor derived cells (**A**). (**C-F**) Expression and relative quantification of the glycosphingolipid GM2 by immunofluorescence: co-immunostaining for the lysosome marker Limp 1 (green fluorescence) and for GM2 (red fluorescence), revealed accumulation of the ganglioside only in differentiated cultures derived from NPC (**D**) and Sandhoff (**E**) patients, but not in healthy donor derived cells (**C**). **F**) The percentage of GM2 expressing cells was assayed in undifferentiated cells and after exposure to the neural inductive media (5 days in N2 medium + 48 h in N3 medium, see methods), and compared to cells obtained in the same way but from a patient affected by Sandhoff disease, a specific GM2 gangliosidosis. No GM2 accumulation was detected in both healthy controls (CTRL, n = 3) and NPC (n = 3) undifferentiated cells, whereas a significant fraction of differentiated NPC (n = 3) and GM2 gangliosidosis derived cells showed accumulation of the glycosfingolipid. At least 400 cells have been counted for each cell line. Data are presented as mean ± SD of 3 independent experiments; one-way Anova test followed by Bonferroni post-test were utilized to compare means between groups. P values less than 0.05 were considered significant. *, **, ****, p<0.05 vs. columns 1, 2 and 4, respectively. The Sandhoff patient was omitted from the statistical analysis.

To determine whether neuronal cells obtained from NPC patients retained the characteristic NPC phenotype, intracellular accumulation of cholesterol and gangliosides was analyzed by filipin staining and immunofluorescence, respectively. As shown in Figure 3, a massive accumulation of unesterified cholesterol was found in the bodies of differentiated cells obtained from NPC patients (Figure 3B). Furthermore, a percentage of these cells accumulated GM2 ganglioside as well (Figure 3D). Interestingly, the percentage of GM2 positive cells obtained applying the neuronal differentiation protocol to hSKIN-MASC from either NPC patients or a patient affected by Sandhoff disease was comparable (Figure 3F). GM2 accumulation was acquired during the differentiation process since this ganglioside could not be detected both in NPC fibroblasts (data not shown) and in undifferentiated hSKIN-MASC (Figure 3C). No accumulation of GM3 was detected both in normal and NPC cells (data not shown).

### Morphologic analysis of neuronal cells

**Figure 4:**
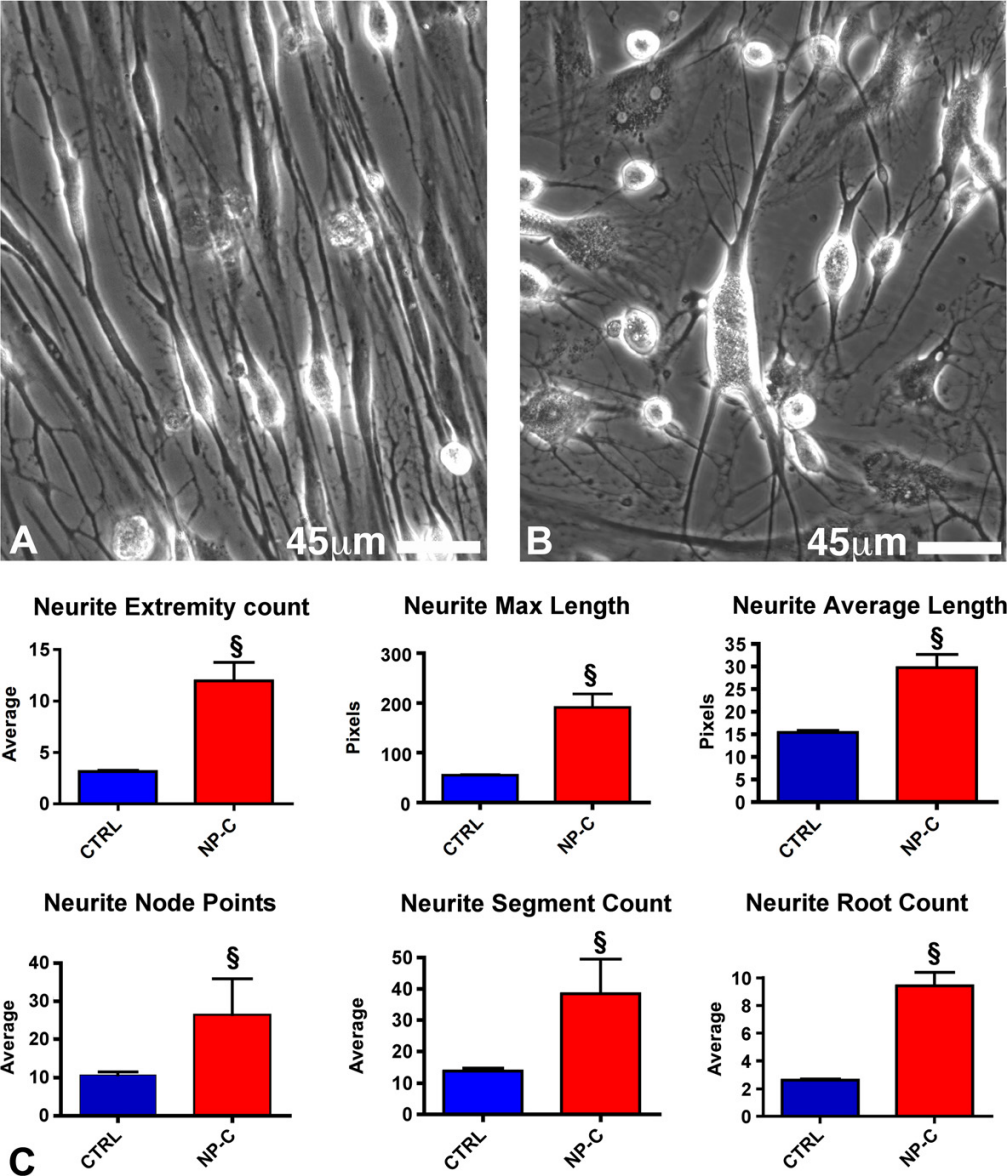
**Morphometric analysis of differentiated cells obtained from hSKIN-MASC of healthy donors or NPC patients.** (**A- B**)**:** Phase contrast image showing the morphological differences between differentiated cells (5 days in N2 medium + 48 h in N3 medium, see methods) of a healthy donor (**A**) and a NPC patient (**B**). Specifically, NPC differentiated cells were characterized by distortion of neuron shape, ectopic neurites and meganeurite formation. (**C**)**:** Quantitative analysis of neuronal morphological parameters linked to neurite outgrowth as evaluated by a specific Image analysis software on MAP-2 stained cells. Data are presented as mean ± SD of 3 independent experiments; §, *p*<0.01 vs healthy donors (CTRL).

As mentioned above, NPC differentiated cells presented a morphology that was clearly different from normal cells. Therefore, in order to quantify the morphologic differences observed, we analyzed the neurite maximal and average length, neurite extremity, segment and root count and neurite node points, using the Neurite outgrowth image program on cells that stained positive for MAP-2. Statistically significant differences were found for all the analyzed parameters between normal and NPC neuronal cell. As shown in Figure 4, NPC differentiated cells presented longer neurites and a greater number of neurite extremities, segments, roots and node points.

## Discussion

The remarkable advances in the ability to obtain stem cells from different tissues, including skin (reviewed in [13,14] and [23]), which are able to differentiate into several cell lineages, offer the possibility to generate cells with neuronal characteristics from easily accessible sources.

It is conceivable then to develop human cellular models for the study of neurodegenerative diseases harvesting cells from skin biopsies of affected patients and committing them to a neuronal fate, thus paving the way to the study of pathways involved in the development of neurological diseases at the cellular level.

NPC is a genetic severe neurodegenerative disease characterized by the accumulation of cholesterol and other lipids within the late endosomes and lysosomes. Although the neuronal degeneration is the main feature in NPC patients, the molecular pathways linking the lipid accumulation and cellular damage in the central nervous system are largely unknown.

Therefore, we developed a human neuronal model of NPC disease using a method based on the induction of neuronal differentiation of stem cells isolated from skin biopsies or primary cultures of fibroblasts obtained from NPC patients.

Here, we demonstrated that it is possible to isolate clonogenic adult stem cells expressing mesenchymal and pluripotent state associated markers both from skin biopsies and fibroblast cultures. In analogy with the multipotent adult stem cells that we have previously isolated from several adult human tissues, these cells were widely multipotent, being able to differentiate into derivatives of the three germ layers [19,20]. For these reasons we named them hSKIN-MASC. The fact that we could obtain multipotent stem cells only from early passages of fibroblasts suggests that immature progenitors may be retained in those cultures but are lost upon serial expansion *in vitro.*

In particular, cells differentiated to neuronal fate expressed markers of mature neurons (NeuN and MAP2) and displayed morphological features resembling neuronal cells. It is worth to highlight that nestin, a marker of the early ectoderm lineage, was expressed both in neural stem cells and in hSKIN-MASC. These data suggest that hSKIN-MASC may represent a population of cells that share some features with the ectoderm lineage. This observation also suggests a common origin between hSKIN-MASC and Skin-derived Precursors or SKP, a multipotent cell population isolated from human dermis that shows a differentiation potential towards both mesenchymal and neural lineages. SKP are believed to be of neural-crest origin [24] and are retained throughout adulthood, although they decrease with age [25].

A preliminary characterization of the type of neuronal cells generated by this method, suggests that under the experimental conditions described, hSKIN-MASCs differentiated to cholinergic but not to dopaminergic or GABAergic neurons. 

Differentiated cells obtained from NPC patients showed some characteristic features of NPC phenotype. They accumulate unesterified cholesterol and GM2 ganglioside. The accumulation of GM2 ganglioside observed in the differentiated cells but not in fibroblasts or undifferentiated hSKIN-MASC, is consistent with data previously reported in NPC patients and NPC mouse models [10,12,26-29]. However, while these studies reported that NPC neuronal cells also accumulate relevant amounts of GM3 ganglioside, no significant accumulation of this ganglioside was found in our model. Since it has been described that the accumulation of GM2 in the brain precedes the accumulation of GM3 [10], it is possible to hypothesize that the model developed here recapitulates the early stage of the disease.

Furthermore, differentiated cells obtained from NPC patients displayed morphological features that are clearly different from those observed in cells obtained from healthy donors, such as the presence of longer neurites and a greater number of neurite extremities, segments, roots and node points. Interestingly, a distortion of the neuronal shape and an extensive growth of new ectopic neurites have been observed in cortical neurons obtained from human patients or animal models of NPC [29,30]. In addition, although there is no clear loss of cholinergic neurons in NPC mice, it has been shown that in these animals cholinergic neurons display several morphological alterations [31].

The identification of morphological alterations in differentiated NPC cells that seem to recapitulate what has been described in human cortical NPC neurons is certainly fascinating. However, we cannot exclude that this phenotype is secondary to an impaired/altered differentiation of MASC isolated from NPC patients. Indeed, the lower percentage of cells expressing NeuN obtained after differentiation of hSKIN-MASC isolated from NPC patients seems to support this hypothesis. Further studies are planned to dissect the molecular mechanisms involved in this phenomenon.

Recently, two human cellular models of NPC disease have been developed by the specific silencing of NPC1 gene in the SH-SY5Y cells line [32] or in human embryonic stem cells [33]. Both models recapitulate the NPC biochemical phenotype. Although neuronal models obtained through the down-regulation of NPC1 expression in human cells may be useful to analyze some aspects of NPC pathogenesis, they are not suitable for the analysis of the impact of specific mutations on the pathologic phenotype or for testing mutation specific therapeutic strategies.

The model described here offers three main advantages with respect to the models cited above: 1-it was developed directly from patient’s cells and therefore it would be useful to analyze the effect of specific NPC1 mutations within the context of the patient genetic/epigenetic background; 2-it was obtained through the differentiation of cells obtained from accessible sources, such as patients cultured fibroblasts, usually available in many laboratories for diagnostic purposes; 3-it did not involve the forced expression of transgenes in target cells, thus avoiding confounding results due to the reprogramming process.

## Conclusion

We have demonstrated that it is possible to isolate stem cells from skin biopsies or fibroblasts in culture and to commit them to a neuronal lineage. Differentiated cells obtained from NPC patients present the main features of NPC disease. Therefore, this model will be useful to study the molecular basis of NPC neurodegeneration and might represent a powerful tool to perform drug screening on cells obtained from NPC patients presenting different genotypes. In addition, the strategy described here may be applied to easily generate human neuronal models of other neurodegenerative diseases.

## Abbreviations

NPC: Niemann Pick C; MASCs: Multipotent adult stem cells; FBS: Fetal bovine serum; DMEM: Dolbecco’s Modified Eagle Medium; BSA: Albumin from bovine serum; PDGF: Platelet derived growth factor; EGF: Epidermal growth factor; bFGF: Basic fibroblast growth factor; VEGF: Vascular endothelial growth factor; IGF1: Insulin-like growth factor 1; HGF: Hepatocyte growth factor; NDS: Normal donkey serum; PAS: Periodic acid Schiff staining; CD31: Platelet endothelial cell adhesion molecule; SMA: Smooth muscle actin; ASA: α-sarcomeric actin; CK: Cytokeratins; MAP2: Microtubule-associated protein 2; GFAP: Glial fibrillary acidic protein; O4: Oligodendroglial protein 4; TH: Tyrosine hydroxylase; DAT: Dopamine transporter; CHAT: Choline acetyltransferase; GAD: Glutamic acid decarboxylase; hMASC: Human multipotent adult stem cells; hSKIN-MASC: Human multipotent adult stem cells derived from skin biopsies and fibroblast cultures; SKP: Skin-derived Precursors.

## Competing interest

The authors declare that they have no competing interests.

## Authors’ contribution

NB: performed the experiments and the statistical analysis. AD: conceived the idea and drafted the manuscript. A B: designed the experiments and helped to draft the manuscript. DC: designed the experiments and helped to draft the manuscript. SR: performed the experiments. SZ: performed the cholesterol and glycolipid analysis. RD: performed the single cell and multipotency experiments. BB: Provide clinical assistance and helped to draft the manuscript. CAB: Coordinate and directed the study. All authors read and approved the final manuscript.

## Supplementary Material

Additional file 1: Table S1 Sequences of oligonucleotides used for real-time PCR. Click here for file

Additional file 2: Figure S1 Mesodermic differentiation of hSKIN-MASC obtained from already established skin fibroblast cultures (hSKIN-MASC). (A-B) Phase contrast images of healthy donor- (A) and NPC patient- (B) derived hSKIN-MASC after exposure to a medium added with IGF-1, bFGF and VEGF. (C-F) Myocyte marker detection: differentiated cells, obtained from healthy donor (C,E) or NPC patient (D,F), express the myocyte specific markers alpha-sarcomeric actin (ASA) (red fluorescence, C,D) and smooth muscle actin (SMA) (red fluorescence, E,F). (G-H) Endothelial cell marker detection: differentiated cells, obtained from healthy donor (G) or NPC patient (H) express CD31 (red fluorescence, G,H). Nuclei are depicted by the blue fluorescence of DAPI staining. (I) Quantitative evaluation of the percentage of cells expressing ASA (i), SMA(ii) and CD31(iii) in cultures from healthy donors (CTRL, n = 3) and NPC patients (n = 3), before (Undiff.) and after exposure to myocyte differentiation induction media (Diff.). At least 400 cells have been counted for each cell line. Data are presented as mean±SD; one-way Anova test followed by Bonferroni post-test was utilized to compare means between groups. *, **, ***, p<0.05 vs columns 1,2 and 3, respectively. Click here for file

Additional file 3: Figure S2 Hepatic differentiation of hSKIN-MASC obtained from already established skin fibroblast cultures. (A-B) Phase contrast images of healthy donor- (A) and NPC patient- (B) derived hSKIN-MASC after differentiation into hepatocytes. (C-F) Hepatic markers detection: differentiated cells, obtained from healthy donor (C,E) or NPC patient (D,F) express the hepatocytes specific markers cytokeratins 8-18-19 (red fluorescence, C,D) and stained positive for the Periodic Acid-Shiff (PAS) staining (pink stain, E,F). Nuclei are depicted by the blue fluorescence of DAPI staining (C, D) or by the blue-stain of hematoxylin (E, F). (G) Quantitative evaluation of the percentage of cells expressing CK in cultures from healthy donors (CTRL, n = 3) and NPC patients (n = 3), before (Undiff.) and after exposure to hepatocytes differentiation induction media (Diff.). At least 400 cells have been counted for each cell line. Data are presented as mean±SD; one-way Anova test followed by Bonferroni post-test were utilized to compare means between groups. P values less than 0.05 were considered significant. *, **, p<0.05 vs column 1 and 2, respectively. Click here for file

Additional file 4: Figure S3 Relative expression of CHAT mRNA in cells derived from healthy donors and NPC patients. The relative abundance of CHAT mRNA were analyzed by real time PCR in cultures from healthy donors (CTRL, n = 3) and NPC patients (n = 3), before (Undiff.) and after neuronal differentiation (Diff.; 5 days in N2 medium + 48 h in N3 medium, see methods). Data were normalized by the expression of GAPDH and expressed as mean as mean ± SD of 3 independent experiments. Click here for file

Additional file 5: Figure S4 Apoptosis in differentiated hSKIN-MASC derived from healthy donors and NPC patients. After induction of neural differentiation (5 days in N2 medium + 48 h in N3 medium, see methods) the levels of apoptosis were evaluated in cultures derived from healthy donors (CTRL, n = 3) and NPC patients (n = 3). Data are presented as mean ± SD of 3 independent experiments. Click here for file

## References

[B1] Patterson M Vanier MT Suzuki K Morris ED Cartsea EB Neufeld EJ Blanchette-Mackie PG Pentchev Scriver CR, Beaudet AL, Sly WS, Valle D Niemann Pick disease type C: a lipid trafficking disorder The metabolic and molecular basis of inherited diseases, Volume 3 2001 VII New York: Mc Graw-Hill 611 634

[B2] Vanier MT Millat G Niemann-Pick disease type C Clin Genet 2003 64 269 281 10.1034/j.1399-0004.2003.00147.x 12974729

[B3] Carstea ED Polymeropoulos MH Parker CC Detera-Wadleigh SD O'Neill RR Patterson MC Goldin E Xiao H Straub RE Vanier MT Roscoe OB Pentchev PG Linkage of Niemann-Pick disease type C to human chromosome 18 Proc Natl Acad Sci U S A 1993 90 2002 2004 10.1073/pnas.90.5.2002 8446622 PMC46008

[B4] Vanier MT Duthel S Rodriguez-Lafrasse C Pentchev P Carstea ED Genetic heterogeneity in Niemann-Pick C disease: a study using somatic cell hybridization and linkage analysis Am J Hum Genet 1996 58 118 125 8554047 PMC1914948

[B5] Davies JP Ioannou YA Topological analysis of Niemann-Pick C1 protein reveals that the membrane orientation of the putative sterol-sensing domain is identical to those of 3-hydroxy-3-methylglutaryl-CoA reductase and sterol regulatory element binding protein cleavage-activating protein J Biol Chem 2000 275 24367 24374 10.1074/jbc.M002184200 10821832

[B6] Naureckiene S Sleat DE Lackland H Fensom A Vanier MT Wattiaux R Jadot M Lobel P Identification of HE1 as the second gene of Niemann-Pick C disease Science 2000 290 2298 2301 10.1126/science.290.5500.2298 11125141

[B7] Boothe AD Kruth HS Weintroub H Stivers J Brady RO A genetic storage disorder in BALB/C mice with a metabolic block in esterification of exogenous cholesterol J Biol Chem 1984 259 5784 5791 6325448

[B8] Maue RA Burgess RW Wang B Wooley CM Seburn KL Vanier MT Rogers MA Chang CC Chang TY Harris BT Graber DJ Penatti CA Porter DM Szwergold BS Henderson LP Totenhagen JW Trouard TP Borbon IA Erickson RP A novel mouse model of Niemann-Pick type C disease carrying a D1005G-Npc1 mutation comparable to commonly observed human mutations Hum Mol Genet 2012 21 730 750 10.1093/hmg/ddr505 22048958 PMC3263988

[B9] Love S Bridges LR Case CP Neurofibrillary tangles in Niemann-Pick disease type C Brain 1995 118 119 129 10.1093/brain/118.1.119 7894998

[B10] Walkley SU Suzuki K Consequences of NPC1 and NPC2 loss of function in mammalian neurons Biochim Biophys Acta 2004 1685 48 62 10.1016/j.bbalip.2004.08.011 15465426

[B11] Vance JE Karten B Hayashi H Lipid dynamics in neurons Biochem Soc Trans 2006 34 399 403 10.1042/BST0340399 16709172

[B12] Zervas M Somers KL Thrall MA Walkley SU Critical role for glycosphingolipids in Niemann–Pick disease type C Curr Biol 2001 11 1283 1287 10.1016/S0960-9822(01)00396-7 11525744

[B13] Beltrami AP Cesselli D Beltrami CA Pluripotency rush! Molecular cues for pluripotency, genetic reprogramming of adult stem cells, and widely multipotent adult cells Pharmacol Ther 2009 124 23 30 10.1016/j.pharmthera.2009.06.003 19545589

[B14] Ratajczak MZ Zuba-Surma E Kucia M Poniewierska A Suszynska M Ratajczak J Pluripotent and multipotent stem cells in adult tissues Adv Med Sci 2012 57 1 17 22515973 10.2478/v10039-012-0020-z

[B15] Kern S Eichler H Stoeve J Klüter H Bieback K Comparative analysis of mesenchymal stem cells from bone marrow, umbilical cord blood, or adipose tissue Stem Cells 2006 24 1294 1301 10.1634/stemcells.2005-0342 16410387

[B16] Anghileri S Marconi S Pignatelli A Cifelli P Galié M Sbarbati A Krampera M Belluzzi O Bonetti B Neuronal differentiation potential of human adipose-derived mesenchymal stem cells Stem Cells Dev 2008 17 909 916 10.1089/scd.2007.0197 18564036

[B17] Seo MJ Suh SY Bae YC Jung JS Differentiation of human adipose stromal cells into hepatic lineage *in vitro* and *in vivo* Biochem Biophys Res Commun 2005 328 258 264 10.1016/j.bbrc.2004.12.158 15670778

[B18] Case J Horvath TL Ballas CB March KL Srour EF *In vitro* clonal analysis of murine pluripotent stem cells isolated from skeletal muscle and adipose stromal cells Exp Hematol 2008 36 224 234 10.1016/j.exphem.2007.09.003 18023524 PMC2553759

[B19] Beltrami AP Cesselli D Bergamin N Marcon P Rigo S Puppato E D'Aurizio F Verardo R Piazza S Pignatelli A Poz A Baccarani U Damiani D Fanin R Mariuzzi L Finato N Masolini P Burelli S Belluzzi O Schneider C Beltrami CA Multipotent cells can be generated *in vitro* from several adult human organs (heart, liver and bone marrow) Blood 2007 110 3438 3446 10.1182/blood-2006-11-055566 17525288

[B20] Cesselli D Beltrami AP Rigo S Bergamin N D'Aurizio F Verardo R Piazza S Klaric E Fanin R Toffoletto B Marzinotto S Mariuzzi L Finato N Pandolfi M Leri A Schneider C Beltrami CA Anversa P Multipotent progenitor cells are present in human peripheral blood Circ Res 2009 104 1225 1234 10.1161/CIRCRESAHA.109.195859 19390058

[B21] Blanchette-Mackie EJ Dwyer NK Amende LM Kruth HS Butler JD Sokol J Comly ME Vanier MT August JT Brady RO Type-C Niemann-Pick disease: low density lipoprotein uptake is associated with premature cholesterol accumulation in the Golgi complex and excessive cholesterol storage in lysosomes Proc Natl Acad Sci U S A 1988 85 8022 8026 10.1073/pnas.85.21.8022 3186703 PMC282346

[B22] Zhou S Davidson C McGlynn R Stephney G Dobrenis K Vanier MT Walkley SU Endosomal/lysosomal processing of gangliosides affects neuronal cholesterol sequestration in Niemann-Pick disease type C Am J Pathol 2011 179 890 902 10.1016/j.ajpath.2011.04.017 21708114 PMC3157170

[B23] Hunt DP Jahoda C Chandran S Multipotent skin-derived precursors: from biology to clinical translation Curr Opin Biotechnol 2009 20 522 530 10.1016/j.copbio.2009.10.004 19896826

[B24] Fernandes KJ McKenzie IA Mill P Smith KM Akhavan M Barnabé-Heider F Biernaskie J Junek A Kobayashi NR Toma JG Kaplan DR Labosky PA Rafuse V Hui CC Miller FD A dermal niche for multipotent adult skin-derived precursor cells Nat Cell Biol 2004 6 1082 1093 10.1038/ncb1181 15517002

[B25] Gago N Perez-Lopez V Sanz-Jaka JP Cormenzana P Eizaguirre I Bernad A Izeta A Age-dependent depletion of human skin-derived progenitor cells Stem Cells 2009 27 1164 1172 10.1002/stem.27 19418448

[B26] Elleder M Jirasek A Smid F Ledvinova J Besley GTN Niemann–Pick disease Type C: study on the nature of the cerebral storage process Acta Neuropathol (Berl) 1985 66 325 336 10.1007/BF00690966 4013680

[B27] Vanier MT Lipid changes in Niemann-Pick disease type C brain: personal experience and review of the literature Neurochem Res 1999 24 481 489 10.1023/A:1022575511354 10227680

[B28] Sleat DE Wiseman JA El-Banna M Price SM Verot L Shen MM Tint GS Vanier MT Walkley SU Lobel P Genetic evidence for nonredundant functional cooperativity between NPC1 and NPC2 in lipid transport Proc Natl Acad Sci USA 2004 101 5886 5891 10.1073/pnas.0308456101 15071184 PMC395893

[B29] March PA Thrall MA Brown DE Mitchell TW Lowenthal AC Walkley SU GABAergic neuroaxonal dystrophy and other cytopathological alterations in feline Niemann-Pick disease type C Acta Neuropathol 1997 94 164 172 10.1007/s004010050689 9255392

[B30] Zervas M Dobrenis K Walkley SU Neurons in Niemann-Pick disease type C accumulate gangliosides as well as unesterified cholesterol and undergo dendritic and axonal alterations J Neuropathol Exp Neurol 2001 60 49 64 11202175 10.1093/jnen/60.1.49

[B31] German DC Quintero EM Liang CL Ng B Punia S Xie C Dietschy JM Selectiveneurodegeneration, without neurofibrillary tangles, in a mouse model of Niemann-Pick C disease J Comp Neurol 2001 433 415 425 10.1002/cne.1149 11298365 PMC3408615

[B32] Rodríguez-Pascau L Coll MJ Casas J Vilageliu L Grinberg D Generation of a human neuronal stable cell model for Niemann-pick C disease by RNA interference JIMD Reports 2012 4 29 37 23430894 10.1007/8904_2011_64 PMC3509875

[B33] Ordonez MP Roberts EA Kidwell C Yuan S Plaisted W Goldstein LS Disruption and therapeutic rescue of autophagy in a human neuronal model of Niemann Pick type C1 Hum Mol Genet 2012 21 2651 2662 10.1093/hmg/dds090 22437840 PMC3363339

