# Supplementary material for: A human neuronal model of Niemann Pick C disease developed from stem cells isolated from patient’s skin
Source: Orphanet J Rare Dis. 2013 Feb 21;8:34. doi: 10.1186/1750-1172-8-34 (PMC3648447; doi:10.1186/1750-1172-8-34)
Supplement: Additional file 1: Table S1 — Sequences of oligonucleotides used for real-time PCR. [file 1750-1172-8-34-S1.docx]

SUPPLEMENTARY TABLE 1

| **GENE** | ***FORWARD PRIMER*** | ***REVERSE PRIMER*** |
| --- | --- | --- |
| ***CHAT*** | 5’CCCTGATGCCTTCATCCA3’ | 5’GTAGGTGGGCACCAGTCTTC3’ |
| ***DAT*** | 5’CCAGCTACAACAAGTTCACCAA3’ | 5’AGAAGCTCGTCAGGGAGTTG3’ |
| ***TH*** | 5’TTGAGGAGAAGGAGGGGAAG3’ | 5’GGATTTTGGCTTCAAACGTC3’ |
| ***GAD*** | 5’TCAAGTAAAGATGGTGATGGGATA3’ | 5’GCCATGATGCTGTACATGTTG3’ |
| ***GAPDH*** | 5’CGACCACTTTGTCAAGCTCA3’ | 5’AGGGGTCTACATGGCAACTG3’ |

Sequences of oligonucleotides used for real-time PCR
