# Supplementary figures and images for: A human neuronal model of Niemann Pick C disease developed from stem cells isolated from patient’s skin
Source: Orphanet J Rare Dis. 2013 Feb 21;8:34. doi: 10.1186/1750-1172-8-34 (PMC3648447; doi:10.1186/1750-1172-8-34)

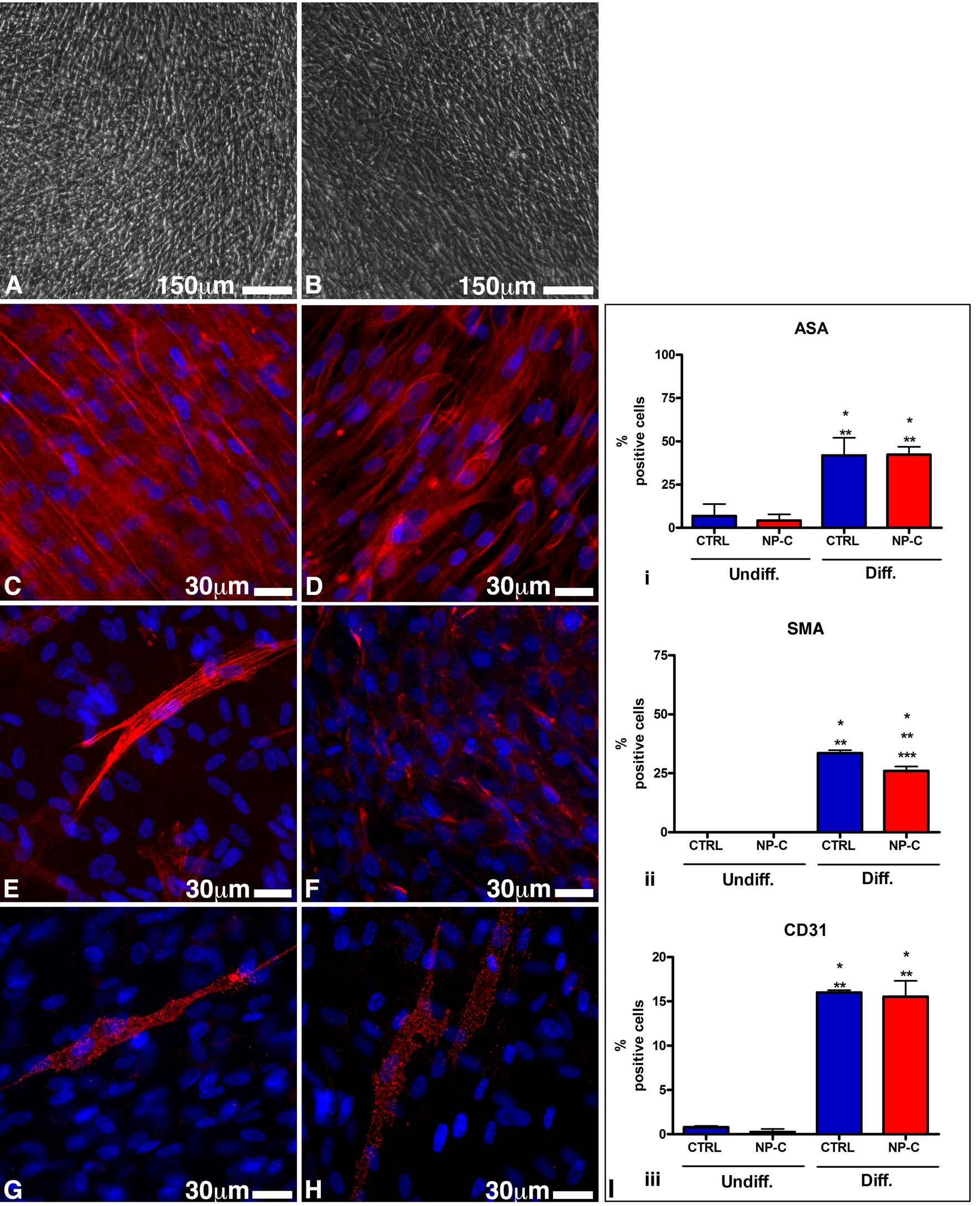

Supplement: Additional file 2: Figure S1 — Mesodermic differentiation of hSKIN-MASC obtained from already established skin fibroblast cultures (hSKIN-MASC). (A-B) Phase contrast images of healthy donor- (A) and NPC patient- (B) derived hSKIN-MASC after exposure to a medium added with IGF-1, bFGF and VEGF. (C-F) Myocyte marker detection: differentiated cells, obtained from healthy donor (C,E) or NPC patient (D,F), express the myocyte specific markers alpha-sarcomeric actin (ASA) (red fluorescence, C,D) and smooth muscle actin (SMA) (red fluorescence, E,F). (G-H) Endothelial cell marker detection: differentiated cells, obtained from healthy donor (G) or NPC patient (H) express CD31 (red fluorescence, G,H). Nuclei are depicted by the blue fluorescence of DAPI staining. (I) Quantitative evaluation of the percentage of cells expressing ASA (i), SMA(ii) and CD31(iii) in cultures from healthy donors (CTRL, n = 3) and NPC patients (n = 3), before (Undiff.) and after exposure to myocyte differentiation induction media (Diff.). At least 400 cells have been counted for each cell line. Data are presented as mean±SD; one-way Anova test followed by Bonferroni post-test was utilized to compare means between groups. *, **, ***, p<0.05 vs columns 1,2 and 3, respectively. [file 1750-1172-8-34-S2.tiff]

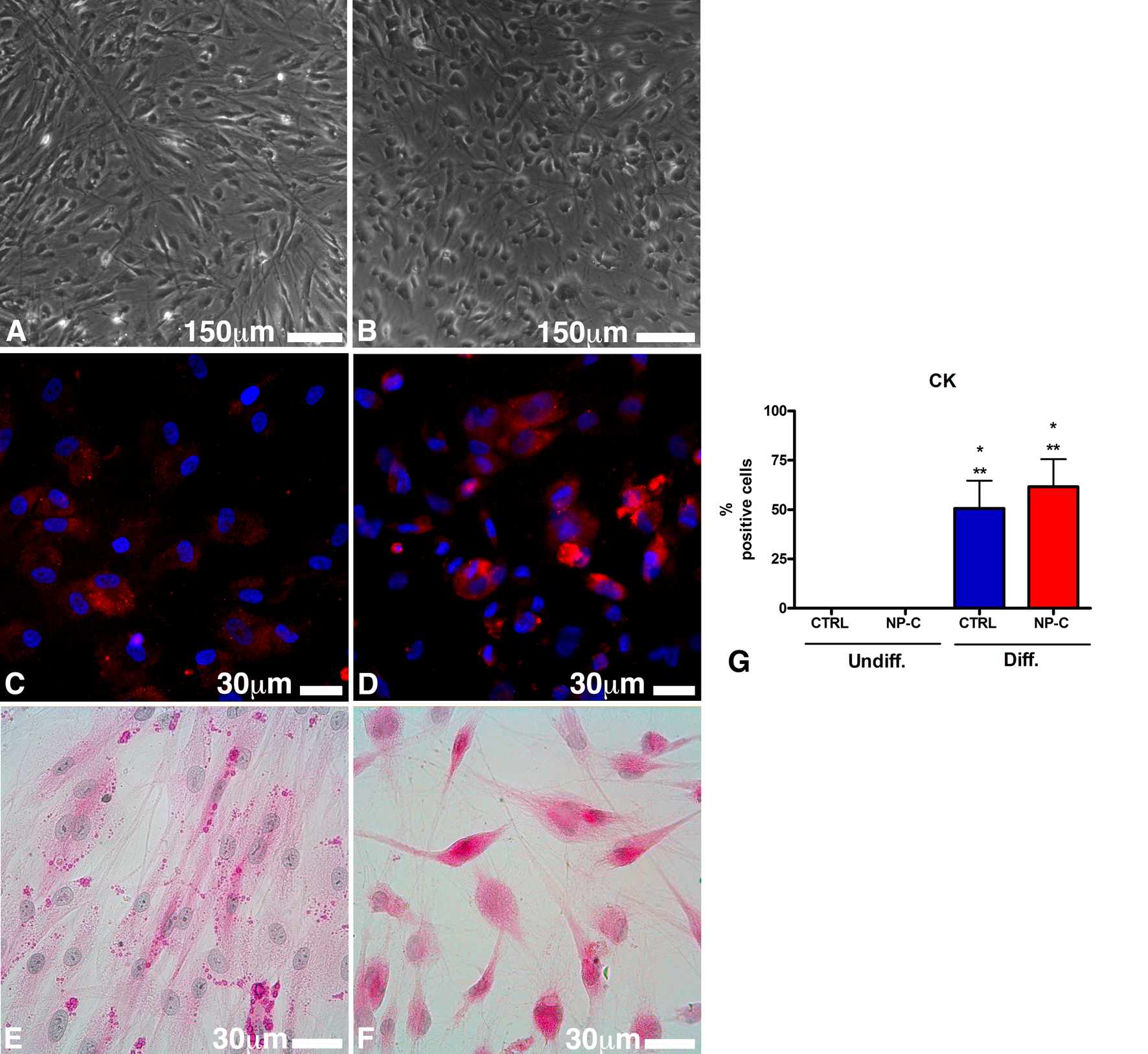

Supplement: Additional file 3: Figure S2 — Hepatic differentiation of hSKIN-MASC obtained from already established skin fibroblast cultures. (A-B) Phase contrast images of healthy donor- (A) and NPC patient- (B) derived hSKIN-MASC after differentiation into hepatocytes. (C-F) Hepatic markers detection: differentiated cells, obtained from healthy donor (C,E) or NPC patient (D,F) express the hepatocytes specific markers cytokeratins 8-18-19 (red fluorescence, C,D) and stained positive for the Periodic Acid-Shiff (PAS) staining (pink stain, E,F). Nuclei are depicted by the blue fluorescence of DAPI staining (C, D) or by the blue-stain of hematoxylin (E, F). (G) Quantitative evaluation of the percentage of cells expressing CK in cultures from healthy donors (CTRL, n = 3) and NPC patients (n = 3), before (Undiff.) and after exposure to hepatocytes differentiation induction media (Diff.). At least 400 cells have been counted for each cell line. Data are presented as mean±SD; one-way Anova test followed by Bonferroni post-test were utilized to compare means between groups. P values less than 0.05 were considered significant. *, **, p<0.05 vs column 1 and 2, respectively. [file 1750-1172-8-34-S3.tiff]

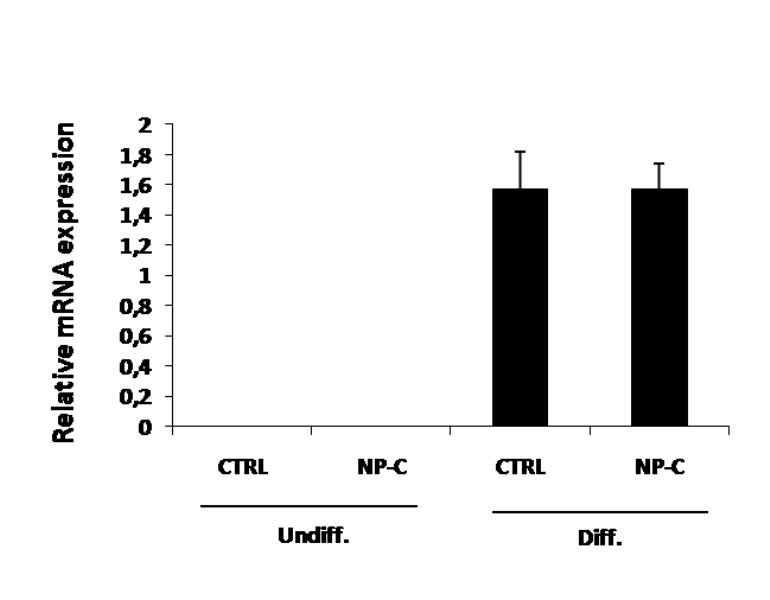

Supplement: Additional file 4: Figure S3 — Relative expression of CHAT mRNA in cells derived from healthy donors and NPC patients. The relative abundance of CHAT mRNA were analyzed by real time PCR in cultures from healthy donors (CTRL, n = 3) and NPC patients (n = 3), before (Undiff.) and after neuronal differentiation (Diff.; 5 days in N2 medium + 48 h in N3 medium, see methods). Data were normalized by the expression of GAPDH and expressed as mean as mean ± SD of 3 independent experiments. [file 1750-1172-8-34-S4.tiff]

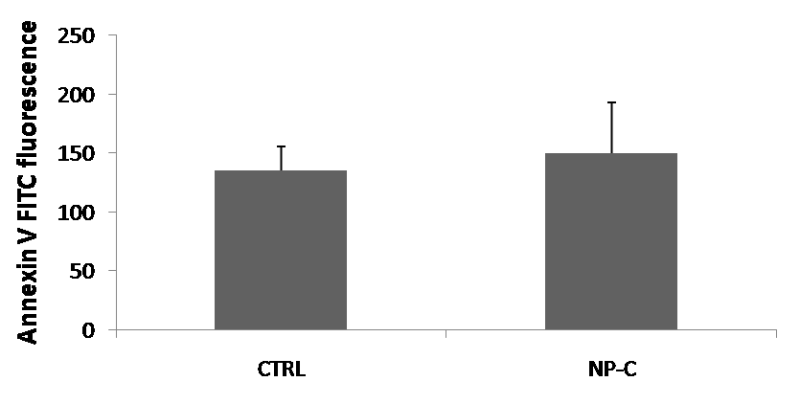

Supplement: Additional file 5: Figure S4 — Apoptosis in differentiated hSKIN-MASC derived from healthy donors and NPC patients. After induction of neural differentiation (5 days in N2 medium + 48 h in N3 medium, see methods) the levels of apoptosis were evaluated in cultures derived from healthy donors (CTRL, n = 3) and NPC patients (n = 3). Data are presented as mean ± SD of 3 independent experiments. [file 1750-1172-8-34-S5.tiff]
